# Unilateral Panuveitis Secondary to Toxoplasmosis in an Immunocompromised Patient: A Case Report

**DOI:** 10.7759/cureus.108599

**Published:** 2026-05-10

**Authors:** Nameer K Rahman, Akshay Narayan, Markus Groppe, Mandeep Bindra, Dimitrios Kalogeropoulos

**Affiliations:** 1 Otolaryngology, Lincoln County Hospital, Lincolnshire Community and Hospitals NHS Group, Lincoln, GBR; 2 Ophthalmology, Stoke Mandeville Hospital, Buckinghamshire Healthcare NHS Trust, Aylesbury, GBR

**Keywords:** extra-pulmonary manifestations of sarcoidosis, ophthalmologic findings of ocular toxoplasmosis, tattoo granulomas with uveitis (tagu), uveitis, retina

## Abstract

Panuveitis is a severe form of intraocular inflammation involving the anterior chamber, vitreous, retina, or choroid, with potential for significant visual morbidity. Differentiating between infectious and non-infectious aetiologies is particularly challenging in immunosuppressed patients, where clinical features may overlap. We report the case of a 54-year-old male patient with rheumatoid arthritis on systemic immunosuppression who presented with unilateral right eye panuveitis. Multimodal imaging was essential in characterising disease activity and monitoring response to treatment. Although initial investigations raised the possibility of ocular sarcoidosis, the overall clinical and imaging findings were more consistent with a presumptive diagnosis of ocular toxoplasmosis. This highlights the limitations of serological testing in immunocompromised individuals. The patient achieved clinical quiescence with residual vitreous changes and stable retinal findings. This case highlights the importance of comprehensive evaluation and multimodal imaging in distinguishing between infectious and inflammatory causes of panuveitis and guiding appropriate management.

## Introduction

Uveitis refers to inflammation of the uveal tract, the middle vascular layer of the eye composed of the iris, ciliary body, and choroid, and may arise from both infectious and non-infectious causes. It is anatomically classified into anterior, intermediate, posterior, and panuveitis, depending on the primary site of inflammation. Anterior uveitis is the most common subtype and affects the iris and ciliary body. Intermediate uveitis primarily involves the pars plana and peripheral retina, while posterior uveitis affects the choroid and/or retina. Panuveitis involves all segments of the uveal tract [1,2].

Uveitis predominantly affects working-age adults and represents a significant cause of preventable visual impairment worldwide, accounting for a substantial proportion of visual acuity decline globally. Despite this burden, the condition remains under-recognised outside specialist settings, and delayed diagnosis carries a meaningful risk of irreversible complications, including cataract, glaucoma, macular oedema, and optic nerve damage [1].

Panuveitis represents a severe form of intraocular inflammation involving the anterior chamber, vitreous, retina, and choroid, and is associated with a higher risk of permanent visual impairment if not promptly diagnosed and managed [2]. Its aetiology is broad and includes infectious causes such as toxoplasmosis, herpes viruses, and tuberculosis, as well as non-infectious inflammatory conditions including sarcoidosis and Behçet disease [3]. These conditions often present with overlapping clinical features, making differentiation particularly challenging in immunosuppressed patients. In this group, serological responses to infectious agents may be attenuated, further complicating diagnostic interpretation.

The variability in presentation across uveitis subtypes complicates both early recognition and therapeutic decision-making, as treatments appropriate for immune-mediated disease, particularly corticosteroids, may be harmful if an underlying infection has not been excluded [1,4]. A multidisciplinary approach integrating clinical findings, multimodal ophthalmic imaging, and targeted systemic investigations is therefore essential to establish an accurate diagnosis and guide management [1]. This report illustrates these diagnostic challenges in an immunosuppressed patient presenting with unilateral panuveitis, where careful and systematic evaluation was required to differentiate between infectious and inflammatory aetiologies.

## Case presentation

A 54-year-old male smoker presented with a one-week history of blurred vision and floaters in the right eye, with associated mild redness but no pain, photosensitivity, or flashing lights. He had a longstanding history of seropositive rheumatoid arthritis, for which he was receiving methotrexate 17.5 mg weekly, leflunomide 20 mg once daily, and folic acid 10 mg weekly. He reported multiple extensive tattoos, the most recently completed approximately nine months before presentation.

Initial examination revealed visual acuity (VA) of 6/7.5 in the right eye and 6/5 in the left. Intraocular pressure (IOP) was elevated at 24 mmHg in the right eye, with a normal contralateral reading of 15 mmHg. Slit-lamp examination demonstrated anterior inflammation (cells 1+, flare 0), keratic precipitates, and 1+ vitritis in the right eye. A working diagnosis of right anterior and intermediate uveitis was made, and a comprehensive serological workup and a chest X-ray were requested. Topical dexamethasone 0.1% (two-hourly followed by gradual tapering), cyclopentolate 1% (three times daily), and dorzolamide/timolol eye drops (twice daily) were commenced.

**Figure 1:**
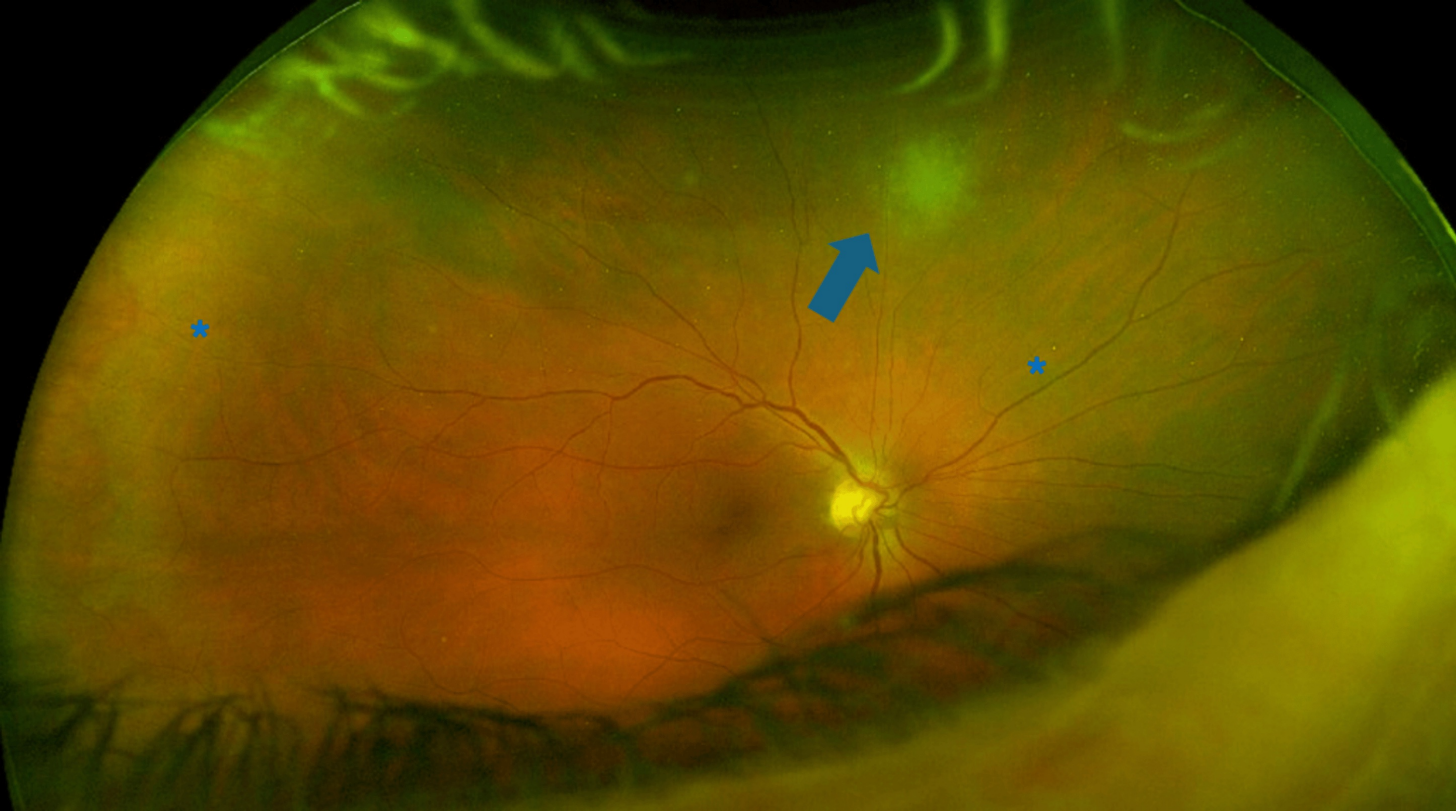
Ultra-widefield fundus imaging demonstrating focal retinitis (blue arrow) and associated retinal vasculitis (blue asterisks) in the right eye The findings are consistent with posterior segment involvement in panuveitis.

At review five days later, VA had deteriorated to 6/15 despite treatment. Dilated fundoscopy and widefield fundus imaging at this stage demonstrated focal retinitis and retinal vasculitis, prompting revision of the diagnosis to right eye panuveitis (Figure 1). Routine bloods, serum angiotensin converting enzyme (ACE), tuberculosis (QuantiFERON-TB Gold Plus (QFT-Plus); QIAGEN N.V., Venlo, Netherlands), human immunodeficiency virus (HIV), and syphilis serology were negative or within normal limits.

Clinical and imaging findings raised the suspicion of toxoplasmosis or sarcoidosis, whereas tattoo-induced uveitis was also considered as an additional differential. Over the next few days, inflammation continued to progress. VA fell further to 6/24, with keratic precipitates and persistent vitritis on examination.

Concerned about the possibility of primary vitreoretinal lymphoma, given the degree of vitritis and the atypical retinal findings, an MRI of the head and orbits was arranged 10 days after presentation, which was normal, reducing concern for lymphoma. With toxoplasmosis remaining the leading presumptive diagnosis in the context of focal retinitis and systemic immunosuppression, oral azithromycin was commenced while *Toxoplasma* and *Borrelia* serology results were pending. Treatment consisted of 1 g on day one, followed by 500 mg daily for three weeks.

**Figure 2:**
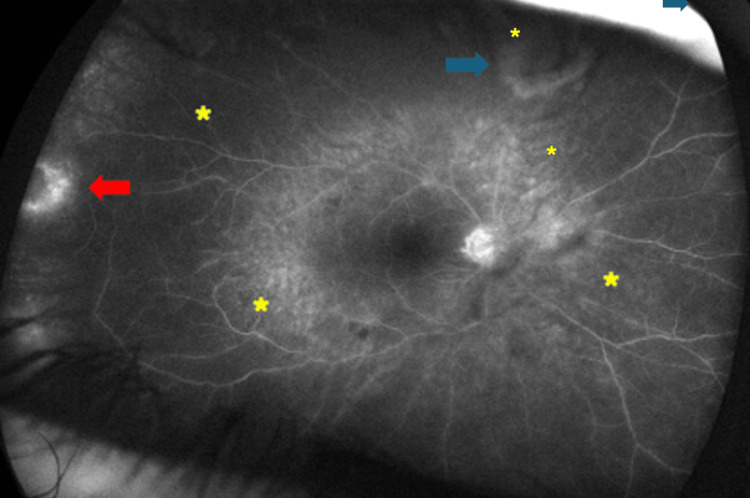
Fluorescein angiography demonstrating active retinal vasculitis and multifocal retinochoroidal lesions in the right eye Fluorescein angiography of the right eye demonstrating vascular leakage and active retinal vasculitis (yellow asterisks). A hyperfluorescent retinochoroidal lesion is observed in the superonasal retina (blue arrow), with an additional temporal hyperfluorescent lesion (red arrow), indicating multifocal involvement.

Fundus fluorescein angiography (FFA) was done 19 days after presentation, which confirmed active vasculitis with peripheral chorioretinal involvement, demonstrating a greater extent of disease than was clinically apparent (Figure 2). In view of the progressive course despite initial treatment, a sub-Tenon injection of triamcinolone 40 mg was administered.

*Toxoplasma* and *Borrelia* serology subsequently returned negative. However, in the context of systemic immunosuppression, this result was interpreted with caution, as both *Toxoplasma gondii* IgG and IgM responses may be reduced or absent in active infection. Although the chest X-ray did not demonstrate any significant abnormalities, chest computed tomography (CT), which had been requested to evaluate for sarcoidosis, revealed numerous bilateral pulmonary nodules, including a cavitating lesion in the right upper lobe. These findings raised the possibility of sarcoidosis or, less likely, metastatic disease. Bronchoscopy and bronchoalveolar lavage were unremarkable. CT-guided biopsy of the pulmonary lesions was performed; critically, the histological findings were consistent with rheumatoid nodules rather than sarcoid granulomata, underscoring the well-recognised but often underappreciated potential for pulmonary rheumatoid disease to closely mimic sarcoidosis on imaging. 

**Figure 3:**
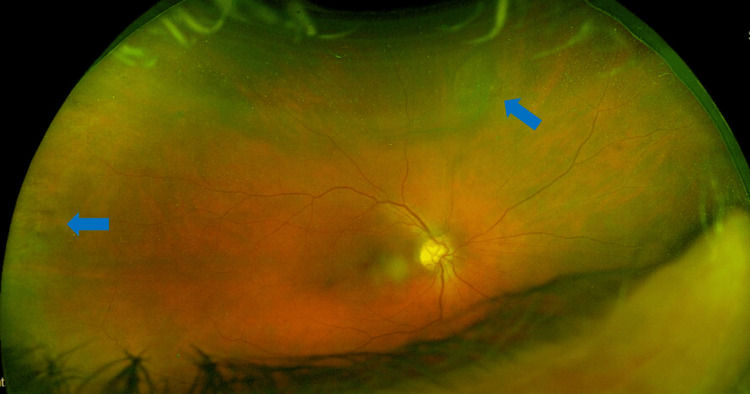
Follow-up ultra-widefield fundus imaging demonstrating resolution of inflammation in the right eye at prior lesion sites (blue arrows)

In the absence of histological evidence supporting sarcoidosis, and given the presence of focal necrotising retinitis with vitritis and vasculitis, the overall clinical picture was considered most consistent with ocular toxoplasmosis despite negative serology. With this working diagnosis established and corticosteroid therapy optimised, the intraocular inflammation responded favourably. Serial imaging demonstrated regression of vasculitis and progressive pigmentation of the retinal lesions, consistent with healing (Figure 3).

**Figure 4:**
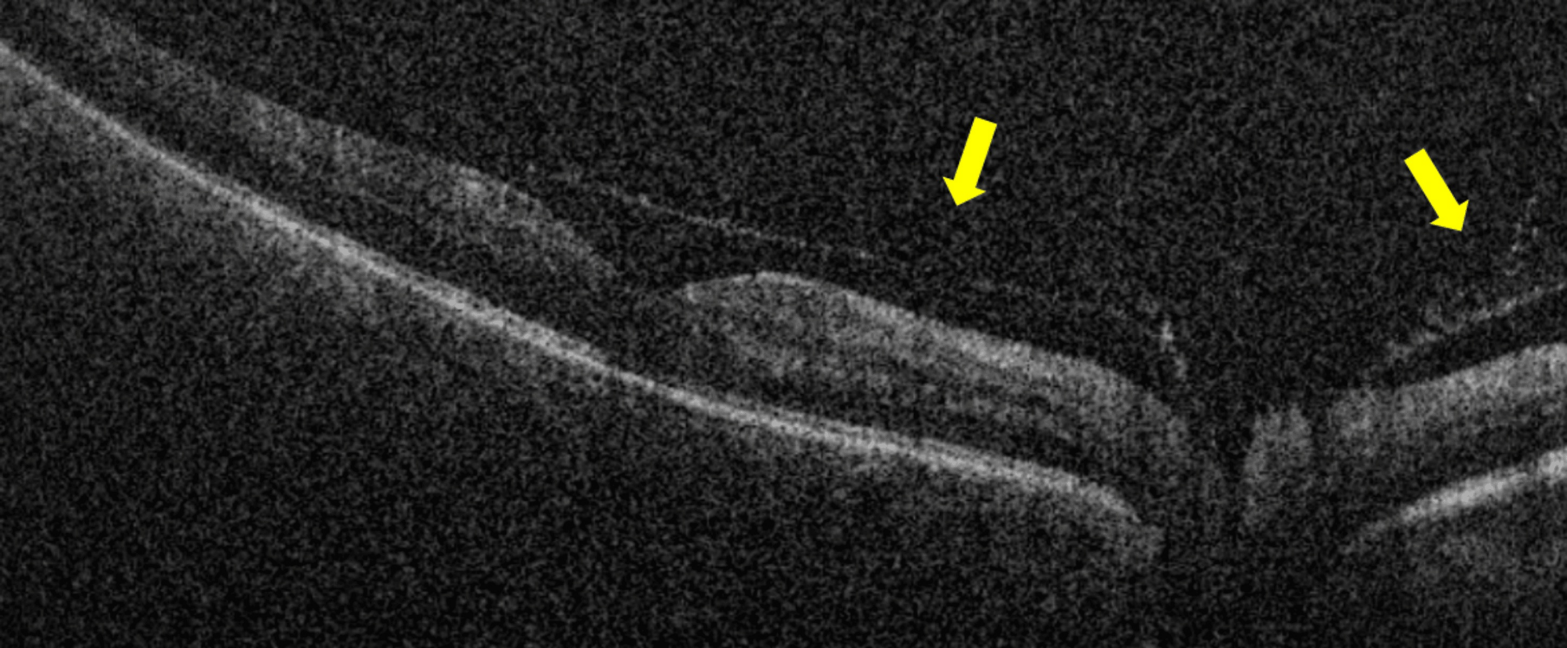
Optical coherence tomography demonstrating preserved macular architecture in the right eye, with no evidence of macular oedema. An incomplete posterior vitreous detachment is also noted (yellow arrows).

Systemic corticosteroids were gradually tapered. At final follow-up, approximately six months after presentation, VA had recovered to 6/7.5 in the right eye. Optical coherence tomography confirmed no macular oedema, and the eye remained clinically quiescent. Residual vitreous debris and early lens changes were noted, and the patient reported persistent floaters, both sequelae of the prior inflammation (Figure 4).

## Discussion

Panuveitis presents a diagnostic and therapeutic challenge due to its heterogeneous aetiology and the overlap in clinical features between infectious and non-infectious causes. In immunocompromised patients, this distinction is particularly complex, as infection and dysregulated immune responses may coexist or mimic one another.

A fundamental principle in the management of retinal vasculitis is early differentiation between infectious and non-infectious aetiologies. While corticosteroid therapy is vision-saving in immune-mediated disease, it may exacerbate untreated infection. Retinal vasculitis is primarily a descriptive diagnosis, supported clinically by perivascular sheathing and confirmed by FFA, where active disease is characterised by vascular wall staining and leakage, often accompanied by areas of capillary non-perfusion or occlusion [4]. FFA is more sensitive than clinical examination alone and frequently reveals a greater extent of disease, thereby influencing both urgency and therapeutic strategy. In this case, FFA demonstrated vasculitis with peripheral chorioretinal involvement, prompting escalation of treatment and close monitoring.

The differential diagnosis of unilateral panuveitis with retinal vasculitis in an immunosuppressed patient is broad and requires a structured approach. Infectious causes include toxoplasmosis, herpetic retinitis, tuberculosis, and syphilis, while non-infectious causes include sarcoidosis, Behçet disease, and systemic vasculitides [4]. Masquerade syndromes, particularly intraocular lymphoma, should also be considered in atypical or treatment-resistant cases. In this patient, the presence of significant vitritis and atypical retinal findings appropriately prompted investigation for lymphoma, with neuroimaging performed to exclude central nervous system involvement.

Ocular toxoplasmosis was initially considered, given the presence of focal retinitis, vitritis, and systemic immunosuppression. Classically, toxoplasmic retinochoroiditis presents as focal necrotising retinitis associated with overlying vitritis and may evolve into a pigmented chorioretinal scar [5]. However, serological testing in this case was not supportive. Importantly, in immunocompromised patients, negative *Toxoplasma* IgG and IgM serology does not reliably exclude active infection, as antibody production may be impaired. It has been demonstrated that ocular toxoplasmosis is typically associated with detectable *Toxoplasma*-specific IgG, and the absence of such antibodies strongly argues against this diagnosis [6]. This principle, however, applies primarily to immunocompetent individuals and should be interpreted with caution in immunosuppressed patients. Despite this, diagnostic uncertainty can persist in immunosuppressed individuals, in whom atypical presentations and altered immune responses are recognised. In such cases, diagnosis may require integration of clinical findings with adjunctive investigations, including intraocular fluid analysis. Polymerase chain reaction (PCR) and assessment of local antibody production have been shown to improve diagnostic sensitivity in challenging cases [7].

Sarcoidosis represents an important non-infectious differential and is a well-recognised cause of granulomatous uveitis. Posterior segment involvement may include vitritis, peripheral chorioretinal lesions, and retinal periphlebitis [4,8]. The revised International Workshop on Ocular Sarcoidosis (IWOS) criteria provide a structured framework for diagnosis by integrating ocular findings with systemic investigations [8,9]. Typical ocular features include mutton-fat keratic precipitates, vitreous opacities, and segmental periphlebitis, while systemic assessment incorporates imaging and, where available, histological findings.

In this case, thoracic imaging revealed multiple pulmonary nodules, raising suspicion for sarcoidosis. However, histological analysis demonstrated rheumatoid nodules rather than sarcoid granulomata, highlighting an important limitation in systemic interpretation. Pulmonary manifestations of rheumatoid arthritis are well recognised and may closely mimic sarcoidosis radiologically. As a result, imaging findings should be interpreted cautiously and in conjunction with clinical and histological data. Following multidisciplinary discussion, the overall clinical, radiological, and biochemical findings were considered most consistent with a sarcoid-like systemic inflammatory process, acknowledging the absence of definitive histological confirmation [10].

However, when integrated with the ocular findings, the absence of confirmatory histology and the characteristic pattern of retinitis and vitritis supported an infectious aetiology, most consistent with ocular toxoplasmosis. Recognition of an infectious aetiology such as toxoplasmosis is critical, as inappropriate immunosuppression may exacerbate disease in the absence of appropriate antimicrobial therapy. Conversely, the concurrent use of antimicrobial treatment and the characteristic pattern of lesion resolution are also consistent with treated ocular toxoplasmosis.

An additional consideration in this case was tattoo-associated uveitis, given the patient’s history of recent extensive tattooing. Although uncommon, tattoo-related immune reactions have been reported to trigger or exacerbate systemic inflammatory disease, including uveitis, likely through delayed hypersensitivity or immune dysregulation mechanisms [11,12]. While not considered the primary driver of disease in this case, it remains an important differential in patients with underlying autoimmune disease.

Multimodal imaging played a central role in both diagnosis and monitoring. Widefield imaging enabled detailed assessment of peripheral retinal involvement and documentation of disease progression, while FFA confirmed the presence and extent of vasculitis. Serial imaging demonstrated regression of inflammation and progressive pigmentation of retinal lesions, consistent with healing. Optical coherence tomography confirmed the absence of macular oedema, correlating with the favourable anatomical and visual outcome.

From a prognostic perspective, retinal vasculitis carries a risk of long-term complications including macular ischaemia, neovascularisation, and recurrent inflammation [4]. Although this patient achieved clinical quiescence, continued surveillance is required. In cases of recurrence or diagnostic uncertainty, early use of targeted intraocular diagnostics may improve aetiological clarity and guide treatment more precisely [6,7].

This case underscores the complexity of diagnosing panuveitis in immunosuppressed patients. The overlap between infectious and inflammatory processes necessitates a systematic and multidisciplinary approach, integrating clinical findings, imaging, and systemic evaluation to arrive at the most plausible diagnosis and optimise patient outcomes.

## Conclusions

Unilateral panuveitis in an immunosuppressed patient represents one of the most diagnostically challenging presentations in ophthalmic practice. Infectious and inflammatory aetiologies may closely mimic one another, and the consequences of misdiagnosis-whether untreated infection exacerbated by corticosteroids or progressive inflammatory disease left undertreated-carry a significant risk of permanent visual morbidity. Importantly, negative *Toxoplasma* serology does not exclude ocular toxoplasmosis in immunocompromised patients and must be interpreted within the broader clinical context.

Accurate diagnosis frequently requires an iterative approach, integrating clinical findings, multimodal imaging, and systemic investigation over time rather than at a single point of assessment. Multidisciplinary collaboration and a willingness to reassess the working diagnosis as new information emerges are essential to achieving diagnostic accuracy and optimising patient outcomes.
